# Altered chronic glycemic control in a clinically relevant model of rat thoracic spinal contusion

**DOI:** 10.1042/BSR20221699

**Published:** 2022-12-23

**Authors:** Kwamie K. Harris, Bradley A. Welch, Allie M. Smith, Yilianys Pride, Bernadette E. Grayson

**Affiliations:** 1 Department of Neurobiology and Anatomical Sciences, University of Mississippi Medical Center, Jackson, MS 39216, U.S.A.; 2 Department of Anesthesiology, University of Mississippi Medical Center, Jackson, MS 39216, U.S.A.; 3 Department of Neurology, University of Mississippi Medical Center, Jackson, MS 39216, U.S.A.

**Keywords:** diabetes, glycemic control, metabolism, spinal cord injury

## Abstract

The lifetime risk for Type 2 diabetes mellitus remains higher in people with spinal cord injuries (SCIs) than in the able-bodied population. However, the mechanisms driving this disparity remain poorly understood. The goal of the present study was to evaluate the impact of a palatable high-fat diet (HFD) on glycemic regulation using a rodent model of moderate thoracic contusion. Animals were placed on either Chow or HFD and tolerance to glucose, insulin, and ENSURE mixed meal were investigated. Important targets in the gut–brain axis were investigated.

HFD consumption equally induced weight gain in SCI and naïve rats over chow (CH) rats. Elevated blood glucose was observed during intraperitoneal glucose tolerance test in HFD-fed rats over CH-fed rats. Insulin tolerance test (ITT) was unremarkable among the three groups. Gavage of ENSURE resulted in high glucagon-like peptide 1 (GLP-1) release from SCI rats over naïve controls. An elevation in terminal total GLP-1 was measured, with a marked reduction in circulating dipeptidyl peptidase 4, the GLP-1 cleaving enzyme, in SCI rats, compared with naïve. Increased glucagon mRNA in the pancreas and reduced immunoreactive glucagon-positive staining in the pancreas in SCI rats compared with controls suggested increased glucagon turnover. Finally, GLP-1 receptor gene expression in the ileum, the primary source of GLP-1 production and release, in SCI rats suggests the responsivity of the gut to altered circulating GLP-1 in the body. In conclusion, the actions of GLP-1 and its preprohormone, glucagon, are markedly uncoupled from their actions on glucose control in the SCI rat. More work is required to understand GLP-1 in the human.

## Introduction

Type-2 diabetes mellitus (T2DM) is a metabolic disorder marked by high fasting and post-prandial circulating blood glucose, increased insulin resistance, and a progressive loss of pancreatic β-cell insulin secretion. The risk for T2DM is increased by obesity such that the high-calorie, high-carbohydrate diets generally consumed in the U.S.A. accelerate disease progression. Obesity develops when food intake disproportionately outweighs energy output, increasing the stores of adiposity in various depots around the body and elevating total body weight. Generally, the overconsumption that comes hand-in-hand with obesity propagates diabetes. Currently, in the U.S.A., 13.0% of all U.S. adults (34.1 million adults aged 18 years or older) have diabetes [1], whereas 42.4% of the U.S. population is burdened by obesity [2]. Predictions indicate that as many as one in three American adults will have diabetes by 2050.

Individuals living with a spinal cord injury (SCI) have a greater lifetime prevalence of T2DM than the general population [3,4]. In fact, in some studies, the overall prevalence of T2DM is three times higher than controls [5]. Partly due to the loss of lean muscle mass through disuse, SCI persons develop visceral adiposity, proven to be the worst prognostic factor for T2DM progression [6,7]. In addition, blood glucose is higher in persons with SCI than in non-injured persons after an oral glucose tolerance test (GTT), which correlates tightly with increased intramuscular fat on MRI [8]. Intravenous GTTs further suggest that SCI persons have elevated insulin and develop resistance to endogenously produced insulin [9]. Thus, the accumulated evidence strongly suggests that glycemic dysregulation, which contributes to metabolic syndrome, is a significant problem in persons with SCI.

Given the human data concerning body weight and glycemic dysregulation in SCI persons, we sought to use a clinically relevant model of spinal injury to investigate the effect of an obesogenic diet on glycemic regulatory control. We performed thoracic level 10 (T10) spinal contusions in male Long Evans rats and compared them to body weight-matched rats naïve to the surgery. Animals were then placed on either standard chow (CHOW) or a 40% butter-fat diet (HFD) for 16 weeks post-injury. We hypothesized that SCI rats would develop glycemic control deficits due to spinal injury that would be exacerbated due to HFD consumption. We tracked body weight and body composition for the study period. In addition, we tested tolerance to glucose, insulin, and a mixed-nutrient meal. We also performed gene expression analysis in the pancreas, hypothalamus, and ileum to understand the effect of diet on glucoregulatory gene expression. Finally, we performed immunohistochemistry on the islets in the pancreas. We report overall changes consistent with dysregulation of glycemic control using this model.

## Materials and methods

### Animal assurance

Procedures complied with the Guidelines for the Care and Use of Laboratory Animals by the National Research Council of the National Academies. All procedures were reviewed and approved by the University of Mississippi Medical Center (UMMC) Institutional Animal Care and Use Committee (IACUC #1469) and the US Army’s Animal Care and Use Review Office (ACURO). The investigators adhered to the laws and regulations of the United States of America Department of Agriculture. All animal studies were performed at the University of Mississippi Medical Center.

### Animals

Long Evans male rats (RRID:RGD_5508398, Envigo, Indianapolis, IN) (250–300 g, approximately 14 weeks old) (Envigo, Indianapolis, IN) were multiple-housed at the UMMC vivarium. They were maintained in a room on a 12/12-h light/dark cycle at 25°C and 50–60% humidity with *ad libitum* access to water and standard chow (#8640, Envigo, 3.0 kCal/g; 17% fat, 54% carbohydrate, 29% protein) for one week before surgery. Rats were then assigned to either the thoracic SCI group or the Naïve group in a counterbalanced fashion based on body weight on the day before the start of surgery. An *N* = 40 (22 and 18 per group, respectively) was used. Larger numbers were used in the SCI group because of the anticipated attrition of the rats. Surgery was performed, and animals were assigned to either continue consuming standard chow or placed on a high-fat diet (HFD, #D03082706, Research Diets, New Brunswick, NJ; 4.54 kCal/g; 40% fat, 45% carbohydrate, and 15% protein) until the remainder of the study which totaled approximately 16 weeks. Our final *N* sizes were as follows: Naïve-chow (*N*=9), SCI-chow (*N*=11), Naive-HFD (*N*=9), and SCI-HFD (*N*=11).

### Surgical procedures

All surgical procedures were performed on deeply-anesthetized animals using 5% isoflurane with a gradual decrease to 2.5%. SCI surgeries were performed as previously described [10–12]. In brief, a laminectomy was performed at thoracic level 10 (T10), and the vertebral column was stabilized at vertebral levels T9 and T11. Using an Infinite Horizon Spinal Impactor Device (Precision Systems and Instrumentation, LLC, Fairfax Station, VA), a moderate contusion was delivered to the T10 spinal cord using 150 kilodynes of force with a 1-s dwell time. The digital trace was inspected to ensure no bone obstruction during the impact. Immediately following the injury, the overlying muscles were sutured, and the skin was securely closed using stainless steel wound clips.

### Post-operative care

Animals received one dose of buprenorphine SR (Sustained Release) (1.0–1.2 mg/kg SQ (ZooPharm, Laramie, WY)) and 72 h later, single-dose buprenorphine for post-surgical pain management (0.025 mg/kg, twice daily for a period of 2 d). Animals also received (1) Naxcel antibiotic (5 mg/kg SQ, Zoetis, NJ) once a day for 5 days, (2) 3–5 ml of saline, twice daily for three days. Each rat’s bladder was manually expressed two to three times per day until the animal recovered independent control to void its bladder. Control of neurogenic bladder function returns in approximately 14 days using this model. Care was generally was discontinued for the bladder when the animal exhibited an already-voided bladder on two consecutive sessions.

### Hindlimb locomotor function assessment

Hindlimb locomotor function was assessed using the Basso, Beattie, and Bresnahan (BBB) open-field locomotor scale [13–15]. BBB scores were initially assessed on day one following injury. Scores achieved on day one post-injury were a mean of 0.045 ± 0.03 for injured animals. Measurements were also made before euthanasia at 16 weeks post-injury. The average scores were 10.86 ± 0.92 for SCI-Chow and 11.91 ± 0.64 for SCI-HFD.

### Body weight and composition

Following surgery, animals were weighed daily for the first seven days and then weekly thereafter. Body lean and fat mass composition was analyzed using Echo Magnetic Resonance Imaging (echoMRI) (EchoMedical Systems, Houston, TX) every 4 weeks for 16 weeks.

### Glucose tolerance test

During post-injury week 13, rats were fasted 6 h after the onset of the light cycle. Baseline tail vein blood glucose was measured using an OnCall Express blood glucometer and corresponding glucose strips (#G135-10D, Acon Laboratories, San Diego, CA). Animals then received an intraperitoneal (IP) dose of 50% dextrose (1.5 g/kg body weight) at time (*t*) = 0 min, and subsequently, blood glucose was measured at 15, 30, 45, 60, and 120 min post-injection. Tail vein blood was collected in EDTA tubes for insulin determination at 0 and 15 min. Insulin measurements were made using an insulin ELISA (#90060, Crystal Chem, IL). Incremental AUC calculation was applied to the blood glucose values to derive an integrated AUC.

### Insulin tolerance test

During week 14 post-injury, fasting tail vein blood glucose readings were obtained following 6 h fasted rats. Rats were given an IP injection of insulin (0.5 U/kg body weight) delivered in 1 ml/kg of saline. Then blood glucose readings were obtained at 15, 30, 45, and 60 min following injection.

### Mixed-nutrient gavage

During post-injury week 15, rats were fasted 6 h after the onset of the light cycle. Baseline tail vein blood glucose was measured, and then animals were given a 5 ml flat dose of Ensure Plus mixed-nutrient meal by intragastric gavage at time (*t*) = 0 min, and subsequently, blood glucose was measured at 15 min post-gavage. Tail vein blood was obtained for the determination of insulin and total glucagon-like peptide 1 (GLP-1).

### Tissue harvest

During week 16 post-injury, rats were 6 h fasted and euthanized by conscious decapitation starting at 2.5 h following the onset of the light cycle. Tissues excised include terminal plasma, ileum, pancreas, and brain. The pancreas was dissected into two portions. Half was homogenized in tri-reagent for 1 min, then flash frozen on dry ice and stored at −80°C until further processing. The other portion was post-fixed in paraformaldehyde (PFA) for histologic assessment.

### Measurements of plasma hormones

Terminal plasma measurements were completed by the Mouse Metabolic Phenotyping Center (MMPC) at The University of Cincinnati, Cincinnati, Ohio (www.uc.edu/labs/mmpc.html). Assays include leptin (#EZRL-83K, EMD Millipore, Billerica, MA), gastric inhibitory peptide (GIP) (#EZRMGIP-55K, EMD Millipore,) total GLP-1 (EZGLP1T, EMD Millipore), C-peptide (EZRMCP2-21K, EMD Millipore) and glucagon (#48-GLUHU-E01, Alpco, Salem, NH). In addition, insulin concentrations were determined using an Insulin ELISA (#90060, Crystal Chem INC., IL). All assays were performed according to the manufacturer’s specifications.

### Paraffin embedding and fluorescent immunohistochemistry

Paraformaldehyde post-fixed pancreas was subjected to standard paraffin-embedding by the UMMC Histology core and then sectioned at 5 µm on to glass slides and processed for slide fluorescent immunohistochemistry.

### Fluorescent immunohistochemistry

Tissue slides were re-hydrated through a series of washes, including xylene, 100%, 95%, 85%, and 70% ethanol for 2 min each. Slides were then washed in 0.1 M potassium phosphate-buffered saline (KPBS). Next, tissue was pre-incubated in blocking buffer (KPBS + 0.4% triton X-100 + 2% normal donkey serum) for 30 min at room temperature, followed by incubation with a cocktail of insulin (1:1000, #i2018, Sigma Aldrich, St. Louis, MO) and glucagon (1:2500, #20076, Immunostar, Hudson, WI) antibodies in blocking buffer overnight at 4°C. Following washes in KPBS, slides were incubated for 1 h in secondary donkey anti-rabbit and donkey anti-mouse antibodies (FITC or Texas Red, 1:500, Invitrogen, Waltham, MA). Fluorescent images were obtained on an Olympus BX60 F5 light microscope with a Leica DFC310 FX camera and Leica Application Suite software version 4.6 (Leica Microsystems, Buffalo Grove, IL).

### RNA extraction

Pancreas, ileum, and brain were harvested and immediately frozen on dry ice, then stored at −80°C until further processing. RNA was isolated with TRIzol® and extracted using a QIAGEN RNeasy Mini Kit (#74104, QIAGEN, Inc, Valencia, CA). RNA content was quantified using the NanoDrop Lite (Fisher ThermoScientific, Waltham, MA). All sample quality was measured using a NanoDrop comparing the 260/280 wavelength and displayed a purity level greater than 2.0.

### Taqman real-time polymerase chain reaction (PCR)

Total RNA was used to transcribe to complementary DNA using an iScript complementary DNA synthesis kit (#1708891, Bio-Rad Laboratories, Hercules, CA). Quantitative PCR was performed on a Step-One Plus Real-Time PCR machine coupled with StepOne Software (v2.3) (Applied Biosystems) using TaqMan inventoried gene expression assays (Life Technologies, Foster City, CA). Samples were analyzed in duplicate, and changes in Ct values from the internal control 60 s ribosomal protein 32 (RPL32, #Rn00820748_g1, ThermoFisher) or GAPDH (#Rn01775763_g1, ThermoFisher) were calculated. The control group average Δ*C*t was made to equal 1. Δ*C*ts of the control group and the experimental groups were then compared, and the fold change was calculated, creating a 2ΔΔ*C*t paradigm. Data were then multiplied by 100 so the control group average Δ*C*t equaled 100.

### Statistics

All statistical analyses were performed using GraphPad Prism version 8.3.1 (GraphPad Software, San Diego, California, U.S.A.). Differences in which three variables (time, injury, and diet) were determined using a three-way ANOVA. Statistical significance was determined with a two-way analysis of variance where there were two variables (injury and diet). Analyses of within-group differences were performed using Student’s *t-*test. Results were considered significant at *P*<0.05, and data are shown as mean ± SEM.

## Results

**Figure 1:**
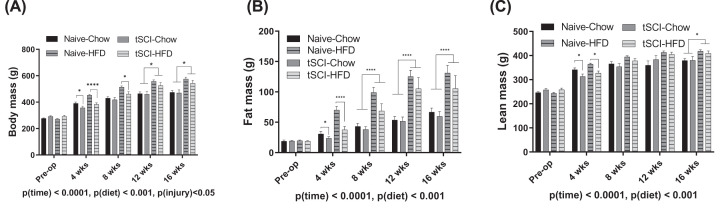
Body weight and composition measurements (**A**) Body mass for naïve and SCI, chow and HFD-fed rats measured before and 4, 8, 12 and 16 weeks after injury. (**B**) Fat mass measured before and 4, 8, 12 and 16 weeks after injury. (**C**) Lean mass for naïve and SCI, chow and HFD-fed rats before and 4, 8, 12 and 16 weeks after injury as measured by echoMRI. Data are presented as mean ± SEM. Statistical significance was determined with three‐way analysis of variance for main effects. Analysis of each time point was determined by two-way analysis of variance for main effects; *P<0.05, ****P<0.0001.

Following SCI, both Chow- and HFD-fed SCI rats lost considerable weight in the first four weeks following injury (Figure 1A). All animals increased in body weight over 16 weeks of the study (Figure 1A). Over the course of 16 weeks, there was a significant increase in weight as a main effect of time and diet, *P*(time) < 0.0001, *P*(diet) < 0.001, with significant differences as a result of *P*(injury) <0.05 (Figure 1A). Differences between Naïve-Chow and tSCI-Chow body weights diminished by 8 weeks post-injury. Body weights of Naïve-HFD and tSCI-HFD rats were significantly different at 4 weeks (*P*<0.0001) and 8 weeks (*P*<0.01) (Figure 1A). By 12 weeks, the major differences in body weight were predicated by diet, with HFD rats significantly heavier than their chow-fed counterparts (Figure 1A). Concerning fat mass, the adiposity of all the animals increased over the time frame, *P*(time)<0.0001 (Figure 1B). HFD-fed rats had significantly increased fat mass over chow-fed rats, *P*(diet)<0.001 (Figure 1B), with no significant difference during the study period as a whole on fat mass by injury (Figure 1B). Finally, lean mass averages increased over time, *P*(time)<0.0001 (Figure 1C), with HFD-fed animals having greater lean body mass over chow-fed animals, *P*(diet)<0.05. There was no effect of injury on lean mass (Figure 1C).

**Figure 2:**
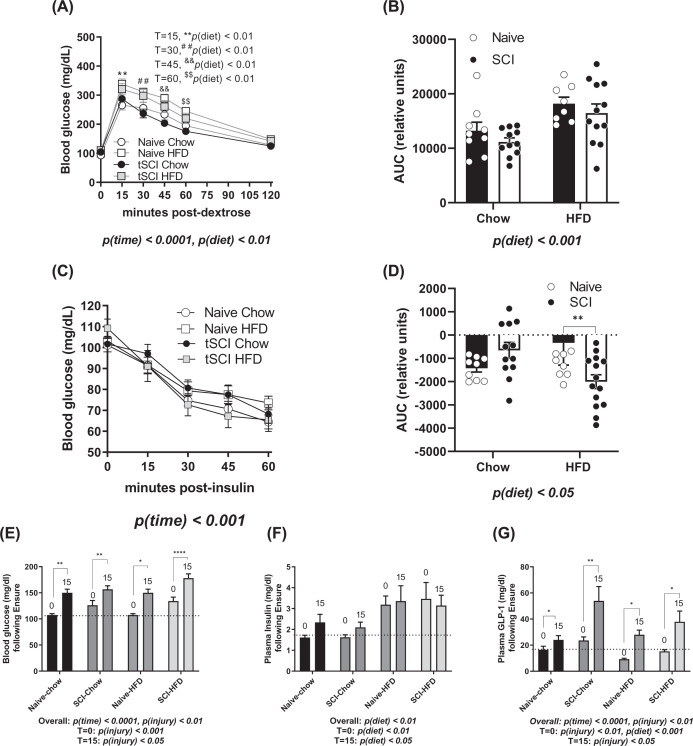
Whole body glycemic measurements (**A**) Blood glucose measurements from the GTT using 1.5 mg/kg dextrose injected intraperitoneally after fasting bleed at *T* = 0. T=15, **P(diet)< 0.01, T=30, ^##^*P*(diet)< 0.01, T=45,^&&^*P*(diet)< 0.01, T=60, ^$$^*P*(diet)< 0.01.(**B**) Area under the curve (AUC) for GTT shown in (A). (**C**) Blood glucose measurements following insulin tolerance test. (**D**) AUC for ITT shown in panel (C). (**E**) Blood glucose at 0 and 15 min following mixed nutrient ENSURE gavage. (**F**) Insulin measurements from blood samples following ENSURE gavage. (**G**) Total GLP-1 levels measured from blood samples obtained before and after ENSURE gavage. Data are presented as mean ± SEM. Statistical significance was determined with three‐way analysis of variance for main effects. *N* = 9–11/group; *P<0.05, **P<0.01, ***P<0.001. Analysis of each time point was determined by two-way analysis of variance for main effects. *P<0.05, **P<0.01. Pre- and post-gavage analyses were performed using Student’s *t*-test; *P<0.05, **P<0.01, ****P<0.0001.

We next tested differences in glucose sensitivity. Following the 6 h fast and injection of dextrose by body weight, blood glucose excursions varied as expected throughout the test with the maximal elevation of blood glucose by 15 min post-injection, *P*(time)<0.0001 (Figure 2A). In total, chow-fed animals had significantly reduced glucose curves in comparison to HFD-fed rats, main effect of diet, *P*(diet) < 0.05, with significant elevations by diet at *T* = 15, 30, 45, and 60, but with no main effect of injury (Figure 2A). This was similarly reflected in the area under the curve (AUC) calculations for the GTT, where HFD-fed animals had higher values than chow-fed animals, *P*(diet) < 0.001 (Figure 2B). After a gap of several days, we next tested insulin sensitivity by performing an insulin tolerance test (ITT). All animals responded to exogenous insulin with reduced blood glucose over time (Figure 2C). Animals that were HFD-fed had more volatile excursions of blood glucose than chow-fed controls, *P*(time) < 0.001, with no impact of diet or injury on the raw values (Figure 2C). When the various starting values were accounted for by performing an AUC for the test, rats fed an HFD had a more robust change in insulin values, *P*(diet) < 0.01 (Figure 2D).

Next, we used a mixed-nutrient meal of ENSURE to induce an acute glycemic response. Blood glucose was elevated in all the animals during the first 15 min following 5 ml intragastric bolus of ENSURE, *P*(time) < 0.0001 (Figure 2E). Baseline blood glucose was elevated in SCI rats compared with naïve, *P*(injury) < 0.001. Elicited blood glucose levels were greater in SCI rats post-gavage than naïve rats, *P*(injury) < 0.05 (Figure 2E). Baseline glucose levels were significantly elevated in tSCI rats in comparison with Naïve, *T* = 0: *P*(injury) < 0.001. Elicited glucose levels were also significantly elevated in tSCI rats compared with Naïve rats, *T* = 15: *P*(injury) < 0.05. Taken together, SCI rats had overall greater glucose levels, *P*(injury) < 0.01. During this experiment, plasma insulin was elevated both at fasting and after 15 min in the HFD-fed animals, irrespective of injury, *P*(diet) < 0.01 (Figure 2F), with elevations for HFD rats at *T* = 0: *P*(diet)< 0.01 and *T* = 15: *P*(diet) < 0.05 independently. Finally, GLP-1 levels were increased in all animals following ENSURE gavage, *P*(time)<0.0001, (Figure 2G). Baseline GLP-1 (*T* = 0) was reduced as a function of HFD *P*(diet) < 0.01, but were overall elevated in SCI rats, *P*(injury) < 0.01.GLP-1 levels were increased at *T* = 15 in tSCI rats in comparison with naïve controls, *P*(injury) < 0.05 (Figure 2G).

**Figure 3:**
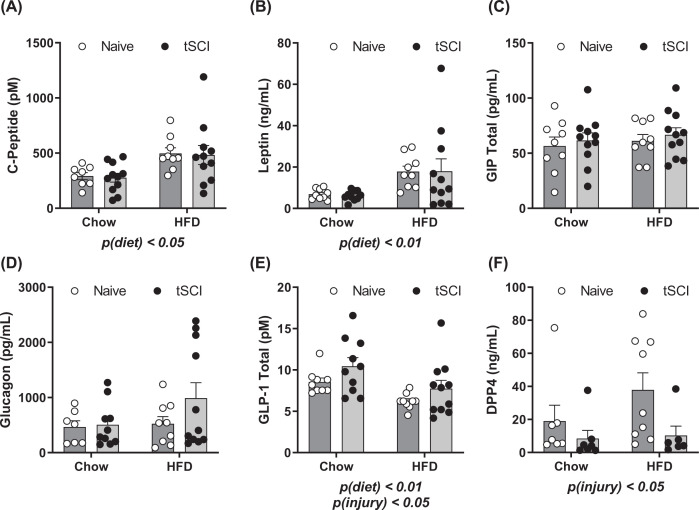
Plasma Hormones measured from terminal plasma 16 weeks post-injury (**A**) C-Peptide (**B**) Leptin (**C**) GIP (**D**) Glucagon (**E**) GLP-1 (**F**) DPP4. Data are presented as mean ± SEM. Analysis of each hormone was determined by two-way analysis of variance for main effects of injury and diet; *P*<0.05, *P*<0.01.

In terminal plasma, fasting plasma metabolic hormones were measured 16 weeks post-injury (Figure 3). C-peptide levels were increased as a result of diet, *P*(diet) <0.05 (Figure 3A). Leptin levels were significantly elevated in HFD-fed animals, *P*(diet) < 0.01, (Figure 3B). No differences between groups were measured in blood levels of gastric-inhibitory peptide (GIP) (Figure 3C) or glucagon (Figure 3D). Total GLP-1, again was reduced as result of HFD consumption, *P*(diet) < 0.01 and elevated as a function of prior SCI, *P*(injury) < 0.05 (Figure 3D). Finally, dipeptidyl peptidase 4 (DPP4) were significantly reduced as a function of prior SCI, *P*(injury) < 0.05 (Figure 3E).

**Figure 4:**
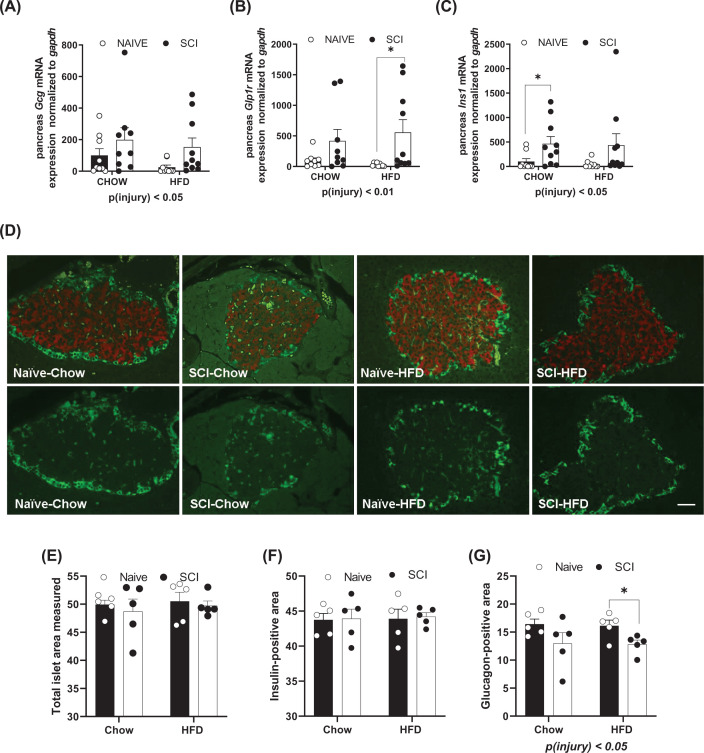
Gene expression and immunofluorescence staining of the pancreas Gene expression by real-time PCR for (**A**) glucagon (*gcg*), (**B**) GLP-1 receptor (*glp1r*), and (**C**) insulin receptor (*ins1*). (**D**) Fluorescent immunohistochemistry in the pancreas for insulin (red) and glucagon (green). Micrometer is 50 μm. Photos taken at 20×. (**E**) Total area of the islet, which includes insulin and glucagon producing cells. (**F**) Total area of the β-cell, which includes insulin producing cells. (**G**) Total area of the α-cells, which includes glucagon producing cells. Data are presented as mean ± SEM. Analysis was determined by two-way analysis of variance for main effects; *P<0.05, **P<0.01. Within-group analyses were performed using Student’s *t*-test. *P<0.05, Scale bar: 25 µms.

We next investigated the members of the gut–brain axis (hypothalamus, pancreas, and ileum) and the gene expression of transcripts involved in glycemic regulation within them. In the pancreas, we measured gene expression for glucagon (Gcg), glucagon-like peptide 1 receptor (*Glp1r*), and insulin 1 (*Ins1*) (Figure 4A–C). All were elevated in the SCI pancreas in comparison with non-injured rats (Figure 4A–C). Pancreas samples were immunostained for insulin and glucagon to determine islet size (Figure 4D). The average size of the islets was calculated by means of dividing the total islet area by the number of islets on the section. There were no differences in the mean islet sizes among groups. There were also no differences in the average area of the largest of the islets in the samples obtained (range of 0.03–0.4 μm^2^). Among the smallest of the islets (range of 0.0007–0.002 μm^2^), HFD-fed rats had significantly larger small islets than Chow-fed rats, *P*(diet) < 0.05. No difference in islet area was measured when normalized to the total area of pancreas stained (Figure 4E). There were no differences in the total immunoreactive area occupied by insulin-positive cells (Figure 4F). However, the total area taken up by glucagon-positive immunoreactivity was reduced in injured rats compared with naïve, *P*(injury) <0.05 (Figure 4G).

Dissecting the medial basal hypothalamus (MBH), we probed for gene targets for glucose-6 phosphate dehydrogenase (*g6pd*), glucokinase (*gck*), and *glp1r* mRNA and found elevations in injured rats in comparison with naïve, *P*(injury) < 0.001 (Table 1). In addition, we also probed for insulin receptor (*insr*) in the MBH and found elevations in injured rats in comparison with controls, *P*(injury) < 0.001, and also an effect of reduced *insr* as a result of HFD-feeding in comparison with chow, *P*(diet) < 0.05 (Table 1). Within the ileum, we found no significant difference in *gcg* expression between groups (Table 1). We observed elevations in *glp1r* gene expression with SCI injury compared with naive, *P*(injury) < 0.05.

**Table 1:** Gene expression of important glycemic markers measured in the medial basal hypothalamus and ileum

Gene	Probe	Naïve-Chow	SCI-Chow	Naïve-HFD	SCI-HFD	Statistics
		Mean ± SEM	Mean ± SEM	Mean ± SEM	Mean ± SEM	
**Hypothalamic expression**						
*g6pd*	Rn01529640_g1	100.0 ± 10.8	180.4 ± 25.6	79.7 ± 7.6	154.4 ± 11.1	*P*(injury) < 0.001
*gck*	Rn00561265_m1	100.0 ± 10.2	149.2 ± 11.1	65.4 ± 4.2	141.4 ± 12.2	*P*(injury) < 0.0001
*glp1r*	Rn00562406_m1	100.0 ± 15.2	162.8 ± 9.8	69.4 ± 9.1	145.2± 13.9	*P*(injury) < 0.001
*insr*	Rn00690703_m1	100.0 ± 9.7	158.6 ± 6.4	75.1 ± 4.2	141.7± 9.1	*P*(injury) < 0.001, *P*(diet) < 0.05
***Ileal gene expression***						
*Gcg*	Rn00562293_m1	100.0 ± 13.6	100.4 ± 7.8	104.0 ± 13.5	107.3 ± 14.8	NS
*glp1r*	Rn00562406_m1	100.0 ± 28.7	195.3 ± 76.2	138.0 ± 23.2	299.6 ± 50.5	*P*(injury) < 0.05, *P*(diet) < 0.05
*dpp4*	Rn00562910_m1	100.0 ± 20.5	182.5 ± 34.2	145.4 ± 35.9	163.0 ± 30.3	NS

Data are presented as mean ± SEM. Statistical significance was determined with two‐way analysis of variance for main effects.

## Discussion

The present study investigated the body-weight and glycemic effects of consuming a normal chow diet or a diet high in saturated fat for 16 weeks following a T10 contusion injury. The data we collected show a potentially novel role of GLP-1 in the pathogenesis of reduced glucose control following SCI.

The changes to body weight and composition following thoracic SCI have been well-established in humans [16] and rats [14,15]. Thoracic SCI results in transient losses to body fat and lean mass in the first 4 weeks that are overtaken by the increased body weight gain due to HFD consumption by both the naïve and tSCI rats after 16 weeks on their respective diets.

SCI rodent studies that report glucose tolerance measures are generally scarce in the literature. We found only one study performed in rats at 1- and 16-week post-SCI [17]. The SCI rats exhibited a reduced AUC for the intraperitoneal GTT, suggesting more sensitive glucose control by the SCI animals [17]. The caveat to this study is that a relatively high concentration of dextrose (2 g/kg body weight) was administered, which could obscure subtle differences during the GTT. On the other hand, in a mouse model of T10 spinal contusion, fasting blood glucose levels 4 h after SCI were significantly elevated over controls and were sustained up to 7 days following injury [18]. This group did not report any other time point, and the elevation probably reflects the traumatic stress of the injury. In our study, we have consistently identified that the Long Evans SCI rats have higher fasting glucose levels and are glucose-intolerant compared with controls when gavaged with dextrose starting at 3 months after injury. Furthermore, the SCI rats persisted in having higher blood glucose levels following a mixed-nutrient meal such as ENSURE.

Using an intraperitoneal injection of glucose, it is not surprising that HFD-fed animals have elevated glucose curves compared with chow-fed animals, irrespective of the surgery. Similarly, when the animals are dosed with insulin based on body weight for the insulin tolerance test, the HFD-fed rats respond most dramatically to produce hypoglycemia. In fact, SCI-HFD rats respond with the most significant reduction, given that they have the lowest lean mass.

The response of endogenous insulin to the glycemic load is different based on diet, such that the greatest insulin response is in the HFD-fed animals. However, paradoxically, the highest GLP-1 levels at baseline and after gavage are in the SCI rats. These data suggest that GLP-1, glucose, and insulin are uncoupled in their traditional responses in the T10-lesioned rat. This is further supported by the fasting hormone levels obtained from terminal blood. Whereas both C-peptide and leptin are elevated due to diet, GLP-1 is paradoxically elevated in SCI rats. For this reason, we further investigated the expression of relevant glycemic genes in other tissues.

GLP-1 is a product of the preproglucagon (*gcg*) gene, specifically expressed in pancreatic islet α-cells, L-cells of the intestinal mucosa, and a set of neurons in the nucleus of the solitary tract (NTS) in the brainstem [19]. *Gcg* is differentially processed within these cells; pancreatic α-cells produce mainly glucagon, while intestinal L-cells and hindbrain NTS neurons produce oxyntomodulin and the bioactive peptides GLP-1 and GLP-2. Thus, GLP-1 is primarily secreted from L-cells in the gut after the ingestion of glucose, fat, or mixed nutrient meals, and plasma GLP-1 increases 2- to 3-fold within 20–30 min and remains elevated for 2 h. GLP-1 is an ‘incretin,’ a gastrointestinal hormone that augments insulin release, inhibits glucagon secretion and gastric emptying and suppresses hepatic glucose production [20]. Plasma GLP-1 is rapidly inactivated to a biologically inactive form by dipeptidyl-peptidase IV (DPP4)—an enzyme ubiquitously found in the body.

Research has exponentially increased regarding the physiological actions of GLP-1 following the FDA approvals of GLP-1 modulating drugs for treating diabetes. These drugs either directly increase GLP-1 actions through GLP-1 agonism or prolong the short availability of endogenously produced GLP-1 by inhibiting its *in vivo* inactivation through DPP4 inhibition. In obesity and T2DM, GLP-1 levels are typically blunted, reducing their beneficial incretin actions and potentiating glucose intolerance [21]. In obese diabetics, fasting GLP-1 levels are significantly reduced compared with non-diabetic obese persons [22]. Further, postprandial GLP-1 secretion in response to oral glucose ingestion is considerably attenuated in obese individuals compared with lean individuals [23], supporting the action of GLP-1 elevating drugs in combatting hyperglycemia. In response to a duodenal infusion of glucose and fat, GLP-1 was not altered in obese subjects, whereas it increased (as expected) in lean individuals [24]. These observations differed from the uncoupling of fasting and elicited GLP-1 levels we observed in T10 contused rats, suggesting a unique role following SCI that has not been studied.

The dysfunction of β cells as diabetes progresses is not well understood. Initially, it was thought that β cell mass was lost due to death, but more recent reports suggest that β cells undergo dedifferentiation [25]. β cells can degranulate and revert to progenitor cell fates [26]. Alternately, the β-cell dedifferentiation may result in conversion into endocrine cell types, of which transcription factor, Fox01, is required to determine the fate of the β cell under these extreme metabolic (excess glucose, obesity) conditions [25]. In our study, the increased glucagon mRNA may result from β-cell transdifferentiation from one endocrine cell type to another [25].

The distribution of relevant receptors and the α-cell mass within the pancreas are distinctively altered following SCI. They appear less influenced in the current study by diet and more actively altered by injury status. Pancreatic α cell mass is reported to be reduced with chronic GLP-1 agonist treatment [27]. Though prolonged drug regimens not surprisingly can affect glucagon production in the pancreas, no examples exist of reduced α cells due to a CNS trauma. Another model in which there are high fluctuations of GLP-1 following nutrient intake does show increased gene expression of GLP-1 receptors in the pancreas chronically in the presence of supraphysiologic GLP-1 [28]; there is also an accompanied reduction in α cell mass [28].

Similarly, in the ileum, we report elevations in GLP-1R expression in the spinal injured rat intestine compared with controls. By mRNA, there are no discernable differences in the pre-prohormone for GLP-1 processing (*gcg*); furthermore, there were no differences in DPP4 gene expression in the ileum. We measured DPP4 levels in plasma and found reduced DPP4 levels. DPP4 is a ubiquitously-expressed cell surface protease that regulates various physiological processes. DPP4 inactivation of GLP-1 predominately occurs in the hepatic portal circulation [29]. Sampling the portal circulation in a clamp experiment within the liver would yield unique information about the activity of DPP4. Further work is needed to identify the cause of elevated DPP4 and the timing of its onset following injury.

Work performed in the human recently suggests that when consuming equal meal sizes, SCI participants report altered perceptions of satiety in comparison with able-bodied controls [30]. In fact, SCI participants have reports of greater satiety given the same meal size [30]. Furthermore, the participants appeared also to have elevated area-under-the-curve for total GLP-1. However, in this human study, only total GLP-1 was reported and not active GLP-1 [30].

## Limitations and future directions

In the current work, we did not *a priori* seek to study GLP-1 and/or DPP4 but rather had open-ended interests in gut hormones; therefore, we did not use protease inhibitors to collect the samples. As a result, using the terminal blood samples we generated, we could not study differences in active or inactive GLP-1, which all require DPP4 inhibitors to be used in the collection media. We identified the phenomenon after the animals were euthanized. We did not investigate time points before 15 weeks post-injury nor know the evolution of these changes in GLP-1 sensitivity. In future studies, the use of the various GLP-1 modulating drugs, both acutely and chronically, may help us understand in the SCI model the nuances of changes in the biological activity of GLP-1. The use of GLP-1 agonists and antagonists and DPP4 inhibitors may help dissect and explore the changes in the GLP-1 system. Using the current data set, we do not know whether GLP-2 or oxyntomodulin are altered, which are processed in parallel with GLP-1. Furthermore, pairing SCI with duodenal catheters to infuse nutrients directly into the intestine may shed light on the functioning of the enteric nervous system and its release of GLP-1. Furthermore, investigating whether the circulating GLP-1 levels influence gastric emptying after thoracic SCI may also help to determine the physiologic role of elevated GLP-1 after SCI.

## Data Availability

Data access is available on request. No mandated data types are included in this manuscript.

## Open Access

Open access for this article was enabled by the participation of University of Mississippi in an all-inclusive *Read & Publish* agreement with Portland Press and the Biochemical Society.

## Abbreviations

DPP4: dipeptidyl peptidase 4
GIP: gastric-inhibitory peptide
GLP-1: glucagon-like peptide 1
HFD: high-fat diet
ITT: insulin tolerance test
SCI: spinal cord injury
